# Indiana

**Published:** 1896-04

**Authors:** 


### COMMENCEMENT EXERCISES.

### INDIANA VETERINARY COLLEGE.

THE annual commencement was held at the College Building, 24 South East Street, Indianapolis, on March 13th. Addresses to the students were delivered by W. S. Tomlin, M.D., and T. L. Armstrong, D.V.S., President.

There were short talks by all of the faculty, and a well-worded response by F. A. Muller, Ph.G., one of the graduates. The following received diplomas: F. A. Muller, Indianapolis, Ind.; H. E. Smock, Indianapolis, Ind.; Charles Regar, Silver Lake, Ind.; W. R. Ramsey, and F. Osborn. Chas. Regar was the winner of the first prize on Surgery, Lameness, and Shoeing, presented by L. A. Greiner, V.S. The first prize consisted of a surgery- and shoeing-case.

